# Sulfur(vi) fluorides as tools in biomolecular and medicinal chemistry

**DOI:** 10.1039/d2ob01891h

**Published:** 2023-01-20

**Authors:** Sabrina N. Carneiro, Samuel R. Khasnavis, Jisun Lee, Todd W. Butler, Jaimeen D. Majmudar, Christopher W. am Ende, Nicholas D. Ball

**Affiliations:** a Department of Chemistry, Pomona College Claremont California 91711 USA Nicholas.ball@pomona.edu; b Pfizer Worldwide Research, Development Groton Connecticut 06340 USA Christopher.amende@pfizer.com; c Pfizer Worldwide Research and Development Cambridge Massachusetts 02139 USA

## Abstract

Recent advances in the synthesis of sulfur(vi)-fluorides has enabled incredible growth in their application in biomolecular chemistry. This review aims to serve as a primer highlighting synthetic strategies toward a diversity of S(vi) fluorides and their application in chemical biology, bioconjugation, and medicinal chemistry.

## Abstract 2

This review highlights key advances in the synthesis and biomolecular applications of sulfur(vi)-fluorides.

## Graphical abstract

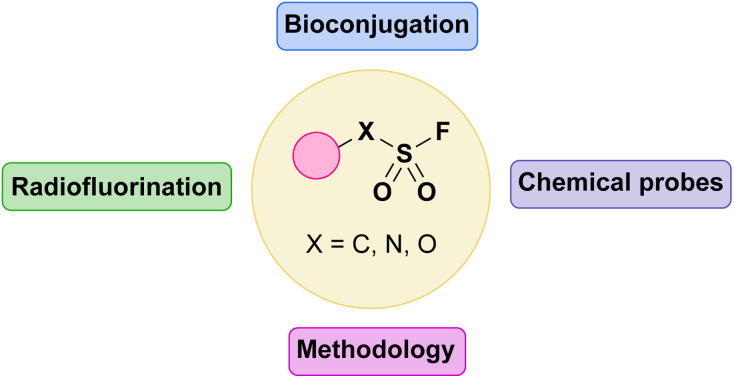

## 1. Introduction

The interaction between synthetic chemistry and biomolecular disciplines has led to incredible discoveries in medicine, elucidation of biological processes, materials for drug discovery, and more. Characterized by their unique redox stability, resistance to hydrolysis, and chemoselectivity organic S(vi) fluorides represent a new class of compounds that have high promise at this interface. Starting from the synthesis of the first organic S(vi) fluorides in the 1920–1930s^1^ and then additional work by Fahrney and Gold^2^ in the 1960s, the utility of S(vi) fluorides saw an expansion in their applications as protease inhibitors and chemical probes. Decades later a foundational paper by Sharpless in 2014 catalyzed the development of more accessible synthetic strategies to make a diverse array of S(vi) fluorides, unlocking a further expansion of their application in biomolecular chemistry.^3^

This review seeks to serve as a source of representative examples of S(vi) fluorides in biomolecular applications. Our aim is to lay out a roadmap for readers to make S(vi) compounds, see key examples of their applications in medicinal chemistry and chemical biology, and identify new challenges and avenues of discovery. While there are excellent comprehensive and focused reviews highlighting either synthetic or biomolecular applications of S(vi)-fluoride compounds,^4–11^ this review aims to provide an integrated prospectus of key examples in organic and biomolecular chemistry across the different classes of S(vi) fluorides. Understanding how to make S(vi) fluorides and seeing how they are applied, will hopefully serve to inspire continued innovation.

## 2. Synthetic strategies toward S(vi) fluorides

Since the re-introduction of S(vi) fluorides by Sharpless, there has been a dramatic increase in synthetic strategies that expand their structural diversity. This section will feature key synthetic methods for the synthesis of an array of S(vi) fluorides.

### 2.1. Sulfonyl fluorides

**Fig. 1:**
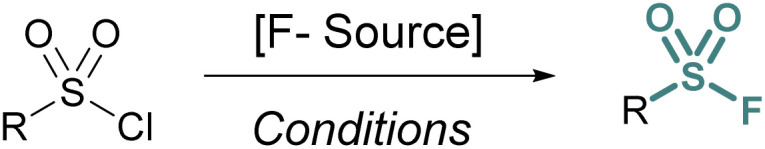
Sulfonyl chloride-to-fluoride conversion with F-sources.

Sulfonyl chloride-to-fluoride conversion is the most common approach to accessing alkyl and aryl sulfonyl fluorides. Since the early 1930s, ammonium, potassium, sodium, and zinc fluoride salts have been used for this process, albeit with limitations in yield.^1^ In an attempt to address this, 18-crown-6 was used in conjunction with potassium fluoride to increase the basicity of the fluoride ion.^12^ While this approach often gave quantitative yields for aryl sulfonyl fluorides, alkyl sulfonyl fluorides were prone to undesired side reactions.^13^ Potassium bifluoride – KFHF^14^ – as an acidic, yet more nucleophilic form of fluoride, helped to mitigate the undesired side reactions.^15^ In these biphasic reactions, it is proposed that nucleophilic fluoride anions (F^−^) are liberated from bifluoride anions (FHF)^−^ by destabilization of fluoride–HF hydrogen bonding interactions at the non-protic organic phase interface.^16,17^ In the presence of sulfonyl chlorides in the organic phase the bifluoride anions deliver an effective F^−^ nucleophile source toward the formation of a wider array of sulfonyl fluorides (Fig. 1).^18,19^

Although sulfonyl chloride-to-fluoride conversion approaches are synthetically convenient, obtaining the corresponding sulfonyl chloride may pose challenges. For example, synthetic methodologies to access sulfonyl chlorides often lack functional group tolerance as they may require harsh reaction conditions. Furthermore, the heightened reactivity of sulfonyl chlorides *versus* other S(vi) compounds can complicate isolation and reduce their benchtop stability. If a commercial or suitably stable sulfonyl chloride starting material cannot be obtained, additional strategies have been developed to prepare sulfonyl fluorides from their chlorinated analogues.^20^

**Fig. 2:**
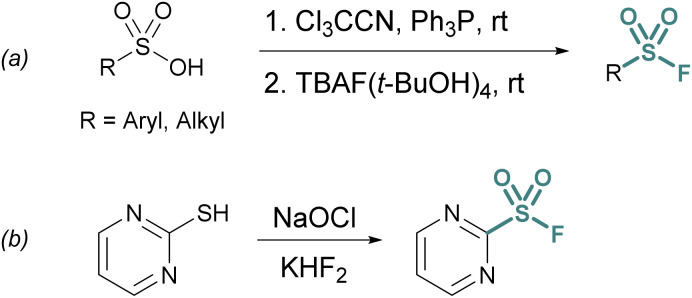
*In situ* generation of sulfonyl chlorides for chloride–fluoride exchange using (a) sulfonic acid activation and (b) oxidation of thiols.

One approach is the *in situ* generation of sulfonyl chlorides. For example, sulfonyl chlorides can be formed *in situ* from stable sulfonic acid precursors using trichloroacetonitrile (Cl_3_CCN) and triphenylphosphine (PPh_3_).^21^ With the addition of a fluoride source (*e.g.*, tetrabutylammonium fluoride (TBAF) (*t*-BuOH)_4_), the transient sulfonyl chloride can undergo Cl^−^/F^−^ exchange to form the corresponding sulfonyl fluoride (Fig. 2a).

Heteroaromatic sulfonyl chlorides can be particularly unstable and therefore pose challenges for isolation and storage. By contrast, heteroaromatic sulfonyl fluorides are markedly more stable enabling the introduction of a heteroaromatic-based sulfonyl group into a myriad of targets. To this end, Wright and Hallstrom developed a mild and inexpensive method to convert heteroaryl thiols to sulfonyl fluorides through oxidative chlorination. Thiols are treated with sodium hypochlorite (NaOCl) to form a sulfonyl chloride *in situ*, followed immediately by Cl^−^/F^−^ exchange using potassium bifluoride (Fig. 2b).^22^

**Fig. 3:**
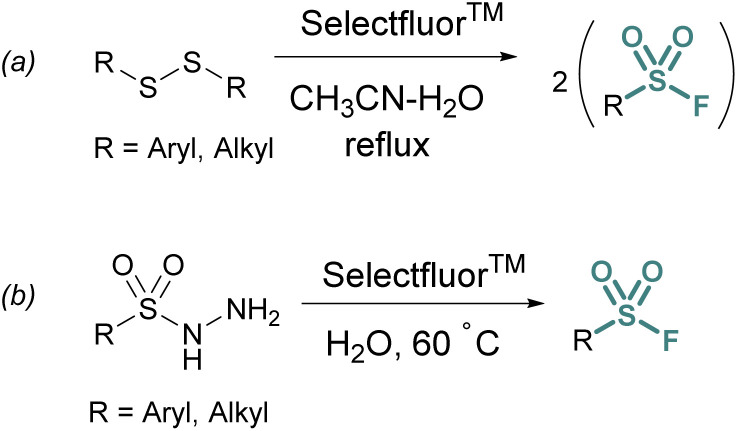
Accessing sulfonyl fluorides with Selectfluor™ from (a) disulfides and (b) sulfonyl hydrazides.

Thiols and disulfides may also be used to generate sulfonyl fluorides without going through a sulfonyl chloride intermediate. Aryl and alkyl sulfonyl fluorides can be generated from their corresponding disulfides using excess Selectfluor™ – a reagent that serves as both an oxidant and electrophilic fluorine source (Fig. 3a).^23^ It is proposed that a sulfur atom is fluorinated to form a sulfonium intermediate, and further oxidized *via* the addition of water.^24^ Selectfluor™ has also found use in the conversion of sulfonyl hydrazines to sulfonyl fluorides in moderate to excellent yields (Fig. 3b).^25^

**Fig. 4:**
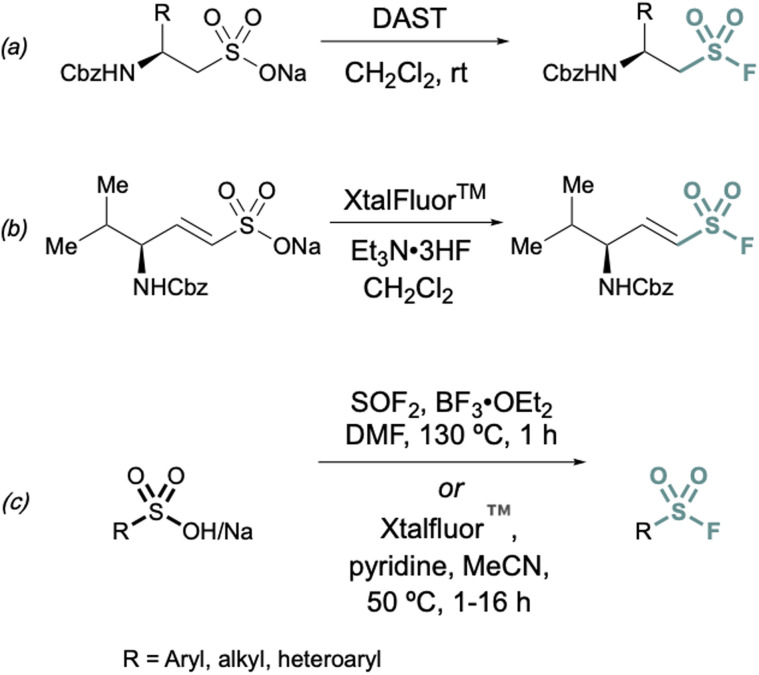
Representative deoxyfluorination reagents (a) DAST, (b) XtalFluor™ and (c) thionyl fluoride (SOF_2_) for converting sulfonate salts to sulfonyl fluorides.

Conversions of alkyl and alkenyl sulfonate salts directly into sulfonyl fluorides have also been reported using deoxyfluorination reagents such as diethylaminosulfur trifluoride (DAST)^26^ and XtalFluor™ ^27^ (Fig. 4). DAST was employed to convert CBz or Fmoc-protected sulfonate salts to aminoethanesulfonyl fluorides (Fig. 4) using alanine, valine, and phenylalanine as the amino acid precursors in moderate to good yields.^26^ Similarly, XtalFluor™ can be used to convert ammonium vinyl sulfonate salts to the corresponding sulfonyl fluoride (Fig. 4b).^27^ Thionyl fluoride (SOF_2_) can also be employed for deoxyfluorination of sulfonic acids salts to make aryl and alkyl sulfonyl fluorides.^28^ In the same report, a complementary method demonstrated the conversion of sulfonic acids and salts to aryl, heteroaryl, and alkyl sulfonyl fluoride using Xtalfluor™.^28^ In contrast to the aforementioned deoxyfluorination reactions with Xtalfluor™ and DAST, this method enables the synthesis of a broader set of sulfonyl fluorides (Fig. 4c).

**Fig. 5:**
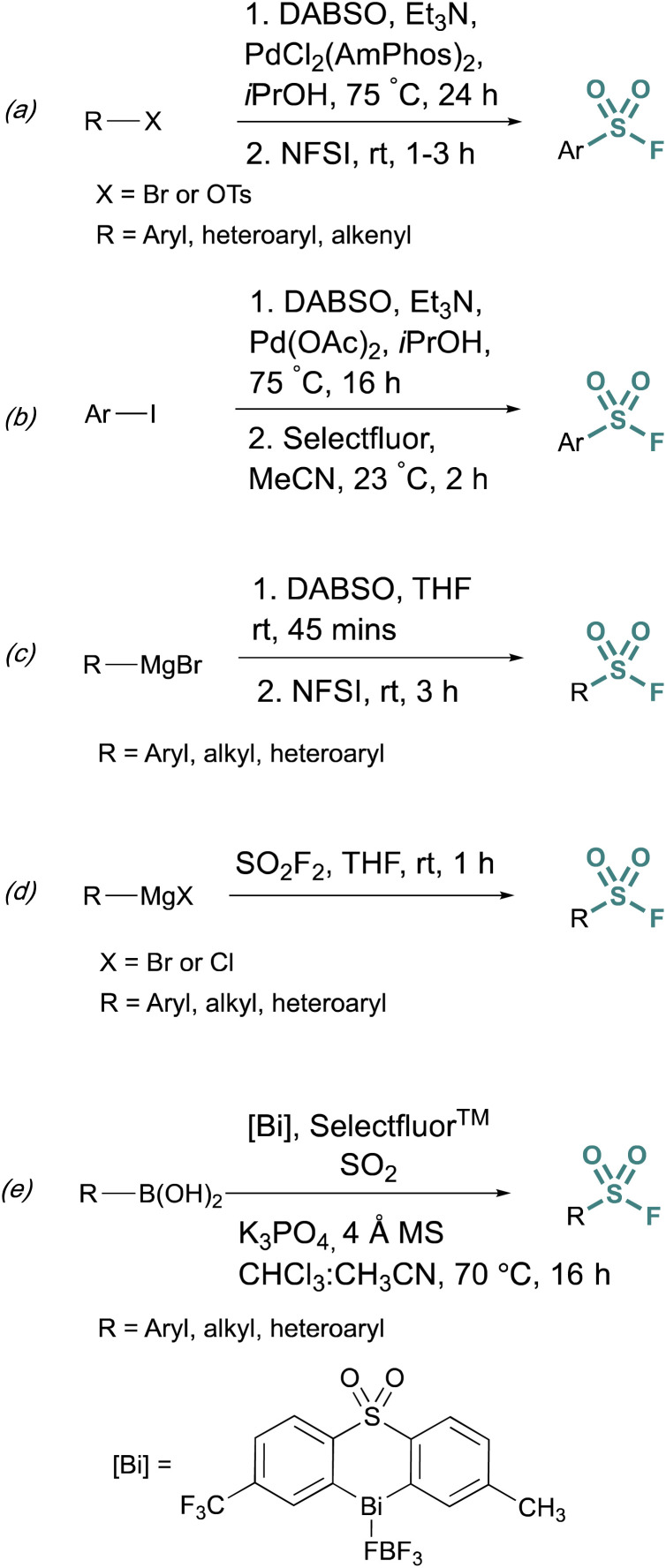
Metalation approaches to sulfonyl fluoride synthesis *via:* (a) aryl bromides, (b) aryl iodides, (c) Grignard reagents and DABSO, (d) Grignard reagents and sulfuryl fluoride, and (e) boronic acids.

Fluorosulfurylation (addition of a SO_2_F moiety) employing transition-metal-catalyzed metalation serves as another alternative to bypass unstable sulfonyl chloride intermediates. These methods use DABSO, a solid DABCO/SO_2_ charge-transfer adduct, that serves as an SO_2_ surrogate. Towards this end, Willis *et al.* developed methods to generate ammonium sulfinate salts in situ from aryl bromides.^29^ and alkenyl triflates using Et_3_N, DABSO, and a palladium catalyst.^30^ In this one-pot, two-step method, *N*-fluorobenzenesulfonimide (NFSI) was used to fluorinate the sulfinate salt, to afford the corresponding sulfonyl fluoride (Fig. 5a). Alternatively, DABSO can be used in conjunction with Grignard reagents and NFSI to generate aryl and alkyl sulfonyl fluorides (Fig. 5c). Ball and coworkers reported a similar palladium-catalyzed method employing Selectfluor™ as the fluorinating reagent (Fig. 5b).^31^ Ball, Sammis, and coworkers demonstrated Grignard reagents can also undergo fluorosulfurylation using *ex situ* generated sulfuryl fluoride (SO_2_F_2_) to access alkyl, aryl, and heteroaryl sulfonyl fluorides *vide infra* (Fig. 5d).^32^ Lastly, following the direct fluorosulfurylation strategy, Cornella and coworkers developed a redox-neutral Bi-catalyzed fluorosulfurylation of aryl and heteroaromatic boronic acids including those with alkenes, alkynes, sulfonamide, and halides, that could be challenging using transition-metal catalysts (Fig. 5e).^33^

**Fig. 6:**
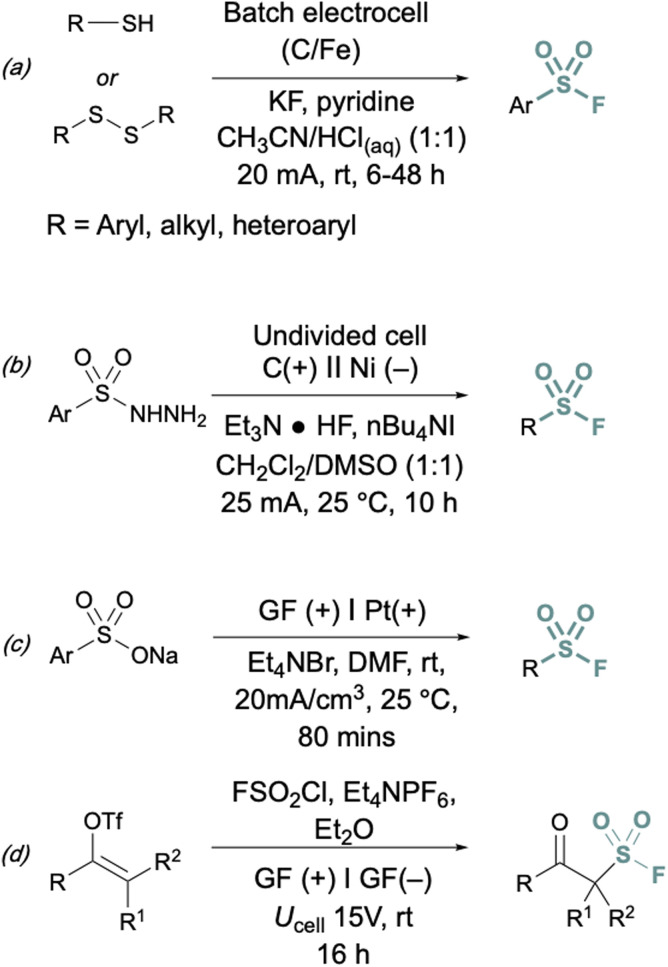
Electrochemical approaches to synthesize sulfonyl fluorides *via*: (a) thiols and disulfides, (b) aryl sulfonyl hydrazines, (c) organic sulfinates, and (d) vinyl triflates.

**Fig. 7:**
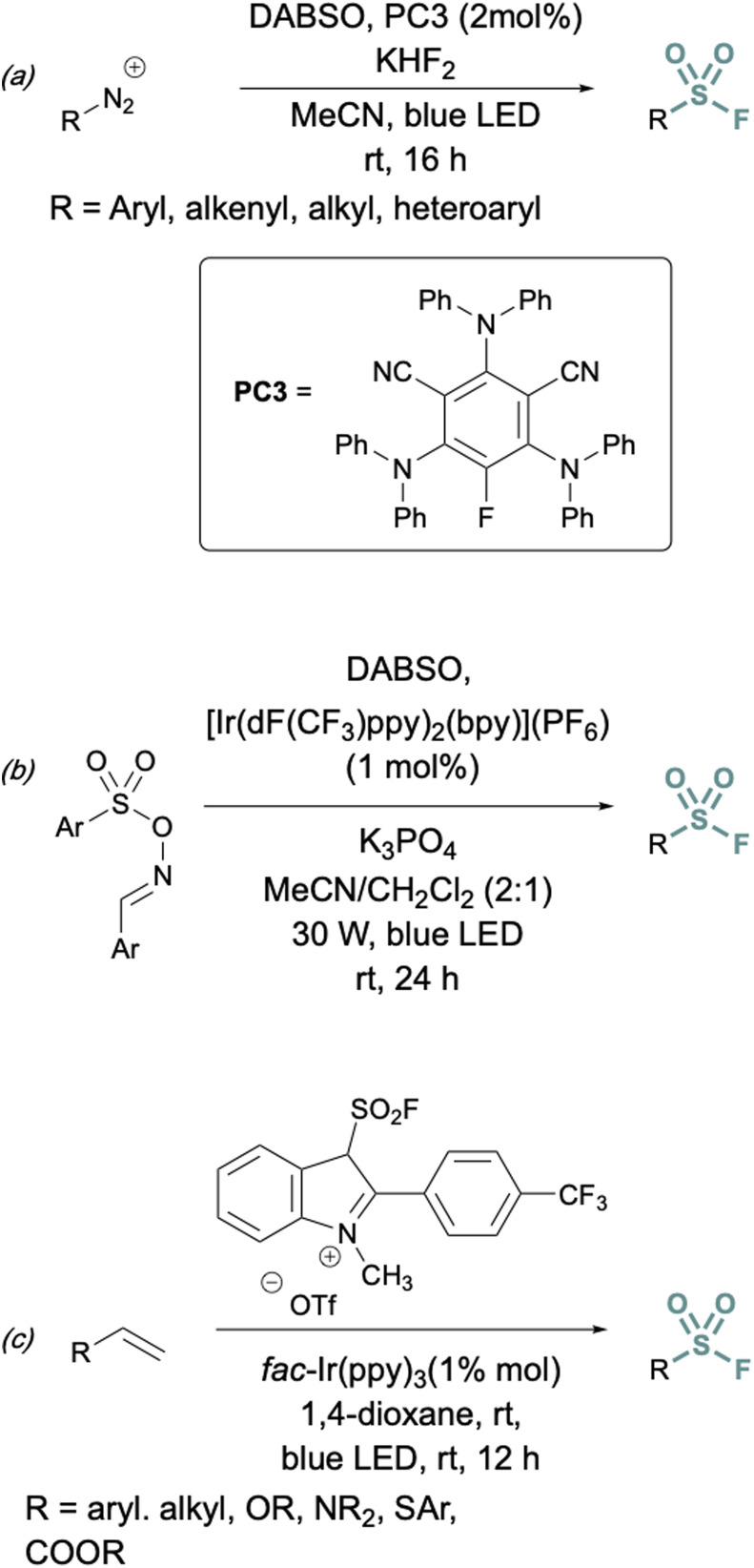
Photochemical approaches to synthesize sulfonyl fluorides using: (a) aryl diazonium salts, (b) carboxylic acid derivatives, and (c) alkenes.

In addition to two-electron strategies, an emerging field in S(vi) fluoride synthesis has centered around electrochemical and photochemical strategies to prepare sulfonyl fluorides.^34–40^ One of the first examples of an electrochemical strategy toward sulfonyl fluorides was reported by Noël and coworkers.^34^ This method involves an oxidant-free, direct conversion of thiols and disulfides to aryl and alkyl sulfonyl fluorides using electrochemical synthesis employing potassium fluoride as a cheap fluoride source. Subsequent electrochemical strategies have converted aryl sulfonyl hydrazines,^35^ organic sulfinates,^36^ and vinyl triflates^37^ to their respective sulfonyl fluorides using various fluoride sources (Fig. 6). Complementary to electrochemical methods, photochemical strategies have also been employed toward sulfonyl fluorides using aryl diazonium salts,^38^ carboxylic acids,^39^ as well as alkenes^40^ as precursors (Fig. 7).

With the diversity of approaches to synthesizing sulfonyl fluorides, it can be challenging to understand where to start. Certainly, which approach is highly dependent on the functional groups of the starting material. For more simple sulfonyl fluoride targets that have functional groups compatible with oxidants, HF^−^, other acids, or FHF^−^, many approaches are suitable (Fig. 2–4). However, there are some strategies that have demonstrated their efficacy toward more complex targets or have starting materials that are more readily accessible. Willis and Ball's DABSO/fluorination fluorosulfurylation approach does provide readily available starting materials in aryl and alkenyl bromides, iodides, and triflates. Additionally, these approaches tolerate a myriad of functional groups including amides, esters, silyl-protected alcohols, and others (Fig. 5). Correspondingly Cornella's Bi-catalyzed fluorosulfurnylation of boronic acids is redox-neutral and could provide a complementary approach to making sulfonyl fluorides that may be sensitive to reduction/oxidation either by metals or oxidizing reagents. Lastly, electrochemical strategies also show promise for sulfonyl fluorides that have complex structures. Huang's electrochemical synthesis of β-keto sulfonyl fluorides from vinyl triflate was demonstrated on derivatives of cholesterol, ibuprofen, tetrahydrogenol, and others.^40^

### 2.2. Fluorosulfates and sulfamoyl fluorides

Synthetic strategies to make more heteroatom-rich S(vi) fluorides like fluorosulfates (ROSO_2_F) and sulfamoyl fluorides (R_2_NSO_2_F) have relied on the direct installation of the fluorosulfuryl (SO_2_F) group. To this end, fluorosulfurylation reagents have been developed to directly install the fluorosulfuryl group to oxygen and nitrogen-based nucleophiles.

**Fig. 8:**
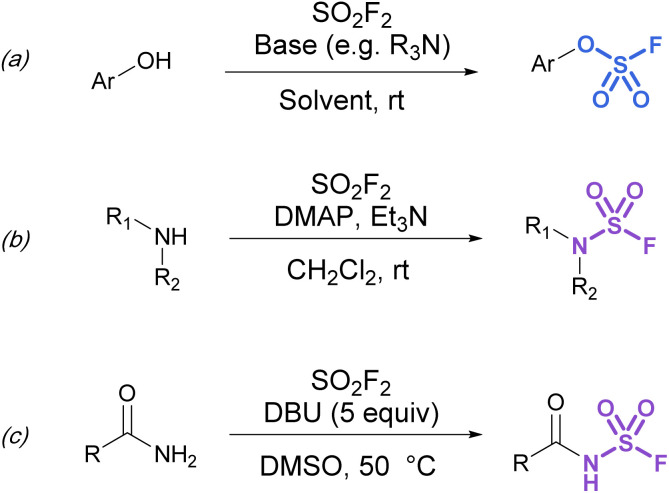
Using SO_2_F_2_ in the fluorosulfurylation of (a) phenols, (b) secondary amines, and (c) amides.

Although fluorosulfates can be prepared using chloride-to-fluoride exchange,^41^ these reactions are not as robust as sulfonyl chloride-to-fluoride conversions. Chlorosulfates can undergo degradation pathways and typically require challenging reaction conditions for synthesis.^3^ Therefore, alternative strategies to access fluorosulfates have been developed. For example, the treatment of phenolates with sulfuryl fluoride gas generates fluorosulfates in high yield.^42^ This method was further improved by Sharpless and is now the most common procedure for efficient fluorosulfurylation of aromatic alcohols (Fig. 8a).^3^ Silyl ethers can also be converted to fluorosulfates under similar reaction conditions using sulfuryl fluoride and catalytic base (not shown).^3^ Likewise, sulfuryl fluoride can be bubbled into a solution of dialkyl-substituted amines with Et_3_N and DMAP to form the corresponding sulfamoyl fluorides (Fig. 8b). However, this method is mainly limited to secondary amines, as primary amines form adducts that can readily undergo fluoride elimination to azasulfenes.^43^ Finally, amides can also undergo *N*-fluorosulfurylation with sulfuryl fluoride, excess DBU, and heating (Fig. 8c).^44^

**Fig. 9:**
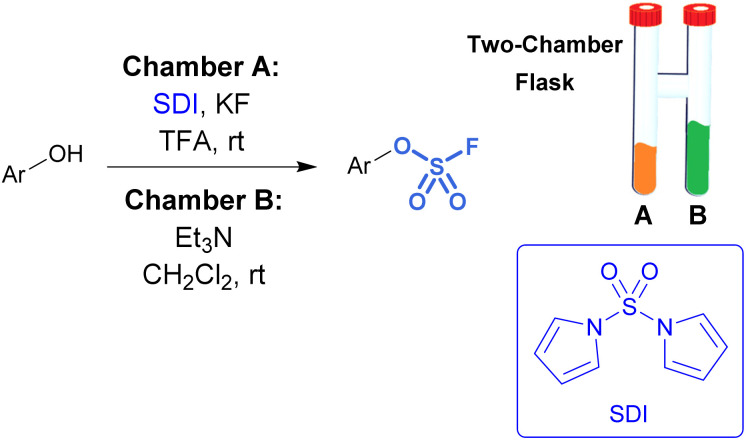
Two-chamber reaction setup for the *ex situ* generation of sulfuryl fluoride gas.

While sulfuryl fluoride is an effective reagent for sulfurylation, it poses notable health risks,^45^ environmental hazards,^46^ as well as the operational inconvenience that comes with working with toxic gases. However, it can be generated *ex situ* or a SO_2_F_2_ surrogate can be employed. For example, De Borggraeve developed a two-chamber *ex situ* protocol to form sulfuryl fluoride for the purpose of aryl fluorosulfate synthesis (Fig. 9).^47^ A controlled amount of SO_2_F_2_ gas is made on demand upon the addition of TFA to 1,1′-sulfonyldiimidazole (SDI) and KF. The generated sulfuryl fluoride gas flows into the second chamber where it diffuses into a solution of phenol, Et_3_N, and CH_2_Cl_2_, resulting in the formation of fluorosulfates. This *ex situ* method was applied to functionalize amino heterocycles, such as ribonucleosides, with sulfamoyl fluoride groups.^48^

**Fig. 10:**
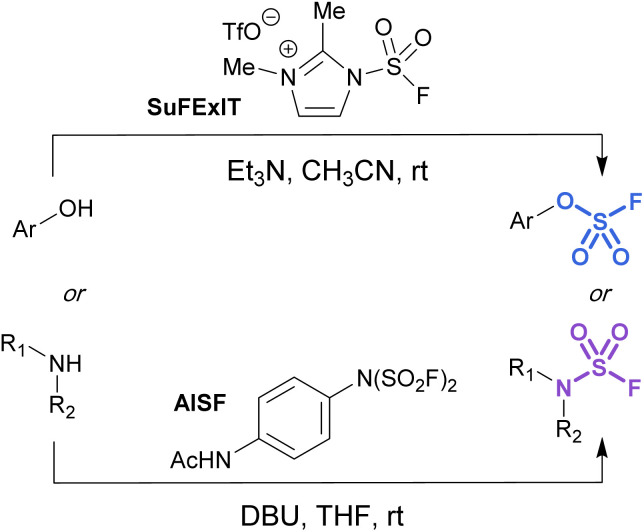
Solid alternatives to sulfuryl fluoride gas for the fluorosulfurylation of phenols and amines.

Alternatively, there are two commercially available SO_2_F_2_ surrogates, a fluorosulfuryl imidazolium salt (SuFExIT)^49^ and [4-(acetylamino)phenyl]-imidodisulfuryl difluoride (AISF),^50^ which are both crystalline solids at room temperature (Fig. 10). Both reagents have been widely utilized in place of sulfuryl fluoride to generate diverse collections of fluorosulfates and sulfamoyl fluorides in high yields. SuFExIT possesses increased reactivity as compared to AISF, with the ability to functionalize anilines with one or two –SO_2_F units, a transformation challenging for AISF. However, AISF has the advantage of being non-hygroscopic and bench-stable at room temperature, as well as not requiring sulfuryl fluoride gas for its preparation.

A remaining synthetic challenge is the synthesis and isolation of alkyl fluorosulfates. Existing chemistry relies on bases to make fluorosulfates. As a result, competing elimination of the fluorosulfate group results in the undesired alkene as the major product. Alternative approaches that eliminate a base – including one-electron processes – could enable the synthesis of alkyl fluorosulfates.

### 2.3. Sulfonimidoyl fluorides, sulfurofluoridoimidates, and sulfuramidimidoyl fluorides

**Fig. 11:**
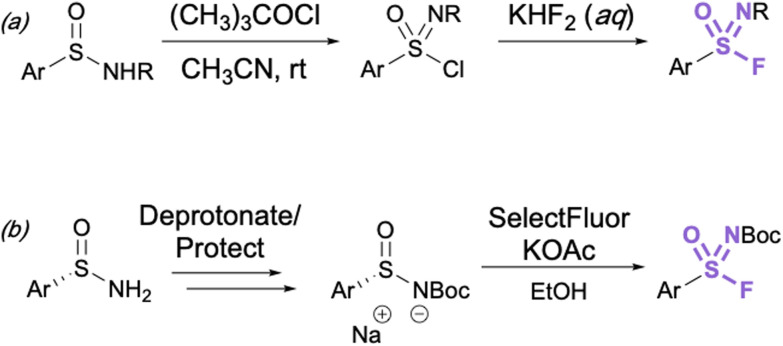
(a) General synthesis of sulfonimidoyl fluorides through chloride–fluoride exchange and (b) preparation of enantioenriched aryl sulfonimidoyl fluorides.

Aryl sulfonimidoyl fluorides (RS = NROF) can be accessed from corresponding sulfinamides *via* a sulfonimidoyl chloride intermediate analogous to that of the sulfonyl chloride–fluoride conversion.^41^ The sulfinamide is oxidized and chlorinated in acetonitrile with *t*-butyl hypochlorite, then an aqueous solution of potassium difluoride is added for the Cl^−^/F^−^ exchange (Fig. 11a).

It is important to consider the consequence of chirality about the sulfur center in the synthesis of sulfonimidoyl fluorides. In this regard, the first stereoselective synthesis of enantioenriched sulfonimidoyl fluorides and their enantioselective conversion to sulfonimidamides has been recently reported (Fig. 11b).^51^ Since excess fluoride ions can racemize enantioenriched arylsulfonimidoyl fluorides, here LiBr was utilized as a fluoride-trapping additive to enable the stereospecific reaction of sulfonimidoyl fluorides with primary and secondary amines (not shown).

**Fig. 12:**
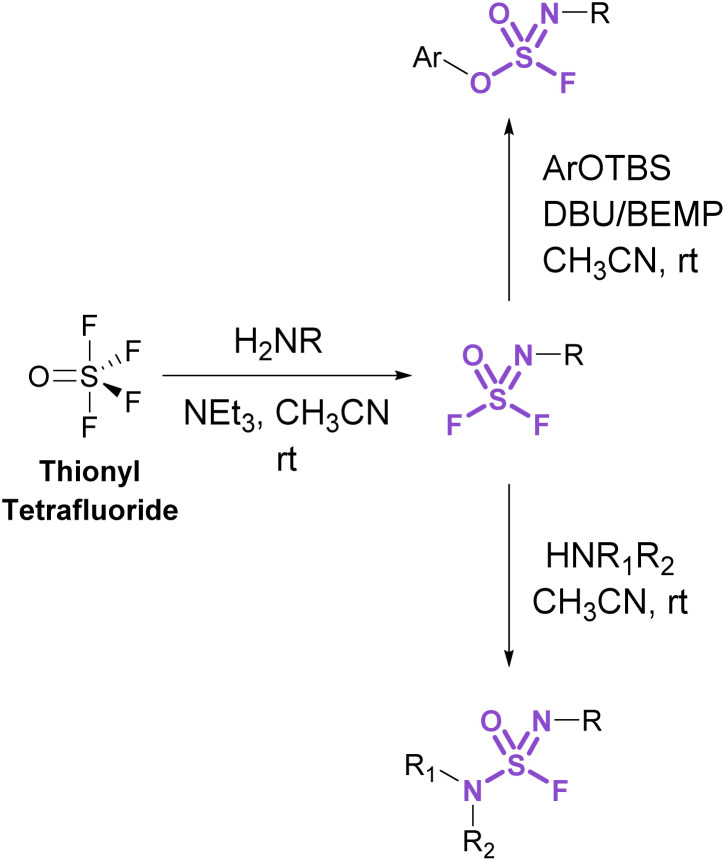
Accessing sulfurofluoridoimidates and sulfuramidimidoyl fluorides using thionyl tetrafluoride.

Sulfurofluoridoimidates (ROS = NROF) and sulfuramidimidoyl fluorides (RNS = NROF) can be accessed from thionyl tetrafluoride (SOF_4_) gas and primary amines *via* an iminosulfur oxydifluoride intermediate (Fig. 12).^52^ Subsequent addition of either secondary amines or aryl silyl ethers converts the difluoride products to sulfuramidimidoyl fluorides or sulfurofluoridoimidates, respectively. Moreover, competition experiments between a mixture of SOF_4_ and SO_2_F_2_ on aminophenols revealed a chemoselective preference for SOF_4_ to functionalize the amine, whereas SO_2_F_2_ modified the phenol. Like sulfuryl fluoride, care should be taken in preparing and handling thionyl tetrafluoride as it is acutely toxic and poses inhalation hazards.^52^

Advances in synthetic strategies for the preparation of these S(vi) fluoride structure classes have also led to their wider use in a variety of chemical biology and medicinal chemistry applications. The following section will feature key examples that demonstrate these applications and aims to serve as a survey of the scientific space.

## 3. Applications of S(vi) fluorides in bioorganic and medicinal chemistry

### 3.1. Sulfonyl fluorides

**Fig. 13:**
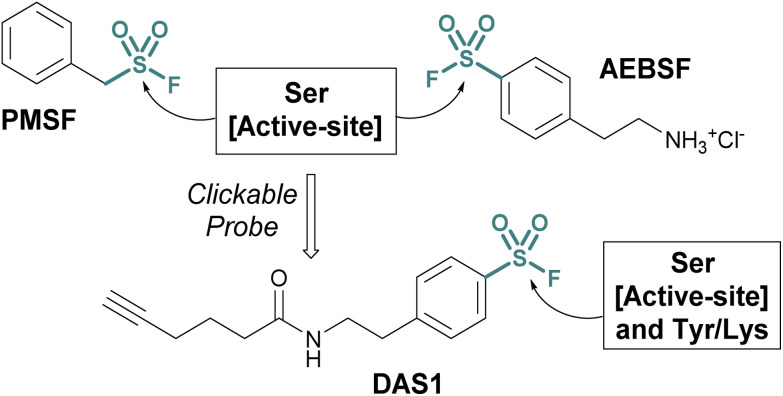
Serine protease inhibitors (PMSF and AEBSF) and the clickable probe DAS1 developed from AEBSF.

Investigations into the biological applications of sulfonyl fluorides largely predate those of other sulfur(vi) electrophiles. Beginning in the mid-1900s, these investigations revealed sulfonyl fluorides act as inhibitors of choline esterases and serine proteases in a manner dependent on their pendant structure and orientation relative to the enzyme's binding pocket.^53,54^ These inhibitors were often found to be irreversible, covalently modifying the enzyme's nucleophilic serine residue.^2,54–56^ For example, phenylmethylsulfonyl fluoride (PMSF), one of the inhibitors tested by Fahrney and Gold in 1963, inactivates serine proteases through active-site serine modification.^2^ PMSF and 4-(2-aminoethyl)benzenesulfonyl fluoride (AEBSF), are still used today as protease inhibitors to suppress proteolytic degradation for cell lysis and protein purification procedures (Fig. 13).

Activity-based probes (ABPs) can be generated from covalent inhibitors by the addition of a reporter group, such as a fluorophore or affinity tag.^57^ This is often accomplished through a bioorthogonal click reaction, such as the copper(i)-catalyzed azide–alkyne cycloaddition (CuAAC). Here, the probe molecule is typically modified with a terminal alkyne, and CuAAC is utilized to conjugate the azide-containing reporter group.^58,59^ Quantifying the engagement of enzymes with the ABP in competition with other inhibitors allows for the determination of the competing inhibitors target enzymes and EC_50_ values *in vivo*. Using this method, an alkyne-tagged AEBSF was demonstrated to covalently label the serine proteases, elastase, chymotrypsin, and trypsin through both in-gel fluorescence analysis and tandem mass spectrometry.^60,61^ However, DiMaggio Jr, and coworkers revealed a challenge using sulfonyl fluoride-based probes for evaluating serine modification, where they demonstrated that the corresponding sulfonyl ester adduct formed on trypsin is hydrolyzed in the workflow and therefore can be challenging to monitor. The authors also developed an isotopic signature strategy that relies on the displacement of the serine-modified sulfonate adduct by 3-bromothiophenol to provide a stable isotopic signature to further aid in understanding probe-modified peptides. Moreover, utilizing the DAS1 probe, additional labeling of Tyr and Lys residues was also observed.^61,62^

**Fig. 14:**
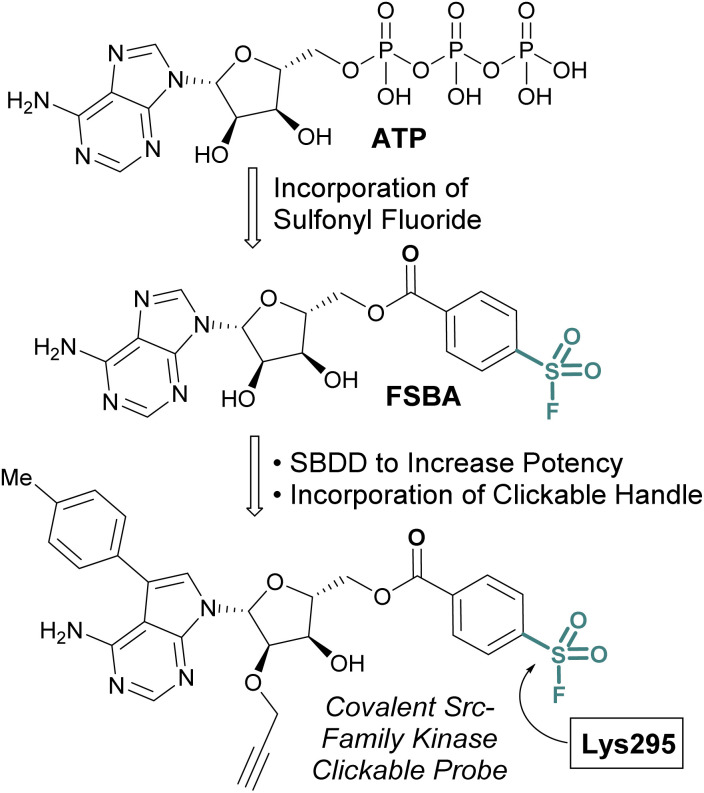
Development of FSBA and an alkyne containing analogue as covalent probes for ATP-binding proteins.

While the sulfonyl fluoride headgroup is necessary for covalent linkage, it must first be appropriately positioned in the proximity of a nucleophilic residue.^61,62^ Structure-based drug design (SBDD) guided modifications to sulfonyl fluoride structures enable the tuning of the reactivity toward specific binding-site residues and the selective modification of particular protein classes.^63,64^ An implementation of this strategy is exemplified with the development of 5′-fluorosulfonylbenzoyl-5′-adenosine (FSBA), an ATP analogue disclosed by Colman and coworkers in 1975, with a sulfonyl fluoride headgroup in place of the triphosphate (Fig. 14).^65^ FSBA covalently inhibits ATP binding proteins (*e.g.* dehydrogenases and kinases), by modifying a conserved lysine residue in the ATP binding pocket. A more potent derivative of FSBA was also developed which incorporated an alkyne handle and was used to measure enzyme-drug occupancy in live cells to identify the selectivity of an FDA-approved tyrosine-kinase inhibitor for kinases within the Src-family (Fig. 14).^66^

**Fig. 15:**
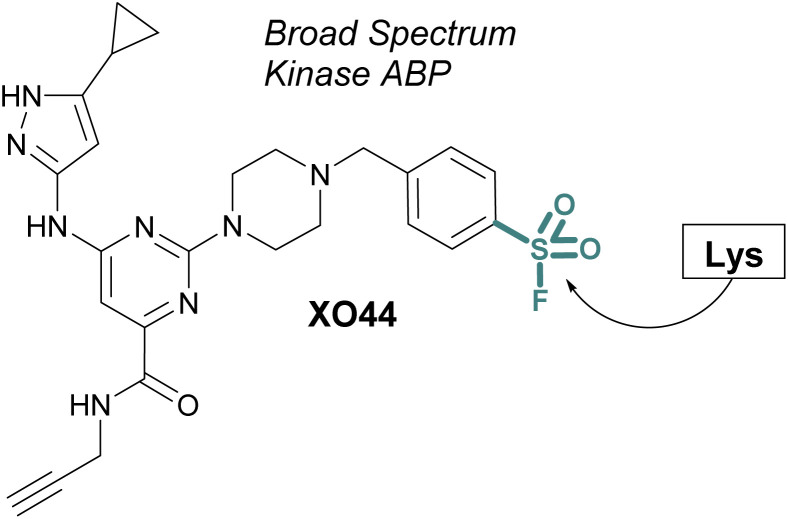
XO44 is an ABP that targets a conserved lysine residue in kinases.

ABPs that cover a broader spectrum of relevant enzymes can be used to understand target engagement across many relevant potential targets of a drug or lead molecule. Toward this end, in 2017 Taunton *et al.*, in collaboration with researchers from Pfizer, used crystal structures of a promiscuous reversible kinase inhibitor (a pyrimidine 2-aminopyrazole scaffold) to design derivatives that placed the sulfonyl fluoride in the proximity of a catalytic lysine residue.^67^ Despite the presence of numerous other solvent-accessible nucleophilic residues, the authors found only the lysine was modified, further showcasing the context dependent mode in which sulfonyl fluorides react with proteins. Evaluating several orientations of the sulfonyl fluoride, XO44 was identified to capture the highest proportion of kinases (133 in total) in live cells, as compared to the other probes evaluated (Fig. 15). XO44 was also used in competition studies to determine the kinase targets and % occupancy of the tyrosine kinase inhibitor, dasatinib. This probe has since found extensive usage in the field of chemical biology and medicinal chemistry due to its high specificity for kinases, broad capture of the kinome, and cell permeability that allows for live cell profiling.

**Fig. 16:**
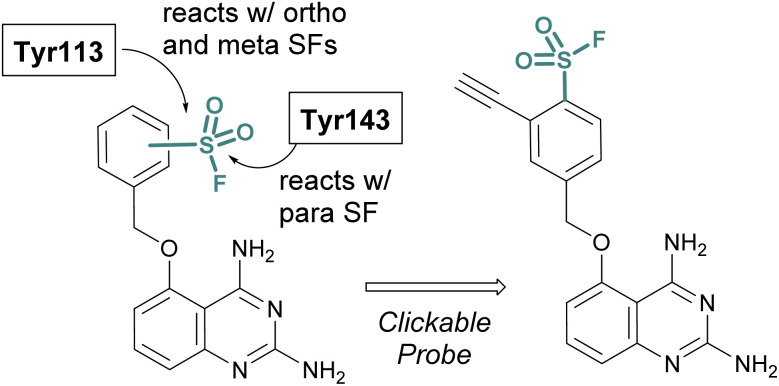
Sulfonyl fluoride containing DcpS probes for targeting non-catalytic tyrosine residues in the ligand binding site.

SBDD can also be used to adapt more selective reversible inhibitors into covalent probes. A differentiating feature of the sulfonyl fluoride headgroup over more commonly used cysteine targeting electrophiles (*e.g.* acrylamides) is their ability to react with any of the nucleophilic amino acid residues (although, some modifications can be labile – *vide supra*).^42^ Toward this end, in 2015 Jones and coworkers at Pfizer published the rational design of a sulfonyl fluoride-modified inhibitor of the mRNA-decapping scavenger enzyme, DcpS to enable target engagement studies in live cells.^68^ Using SBDD, a potent and selective inhibitor of DcpS was modified with sulfonyl fluorides positioned to target tyrosine residues in the binding site. The *ortho* and *meta*-substituted sulfonyl fluoride analogues reacted with Tyr113, whereas the *para*-analogue covalently modified Tyr143 (Fig. 16). The incorporation of a clickable handle allowed the authors to utilize the probe to obtain OC_50_ values of the reversible lead compound in peripheral blood mononucleated cells (PBMCs).

**Fig. 17:**
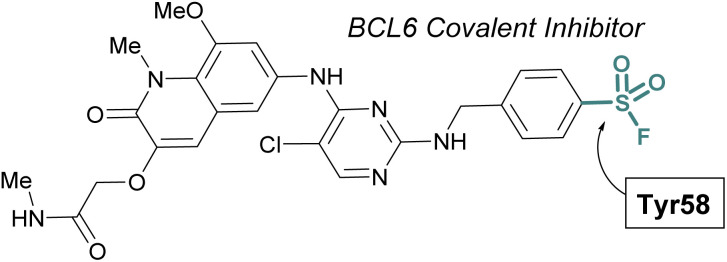
Sulfonyl fluoride containing BCL6 inhibitor targets tyrosine 58 and blocks corepressor protein binding.

The scope of sulfur(vi) fluoride exchange (SuFEx) chemistry extends beyond enzyme active site targeting, as shown in multiple reports that utilize sulfonyl fluorides to inhibit protein–protein interactions (PPIs). For example, using SBDD of known reversible inhibitors of the transcriptional repressor B-cell lymphoma 6 (BCL6), Gray and coworkers demonstrated the targeting of a tyrosine residue with an aryl sulfonyl fluoride moiety (Fig. 17).^69^ This covalent probe effectively inhibited BCL6 in a corepressor peptide displacement assay, thus blocking BCL6's ability to recruit corepressor proteins. It is noteworthy that the sulfonyl fluoride covalent inhibitor was superior to the parent reversible inhibitor in its antiproliferative activity in live cells, a result attributed to the ability of the covalent inhibitor to have a prolonged engagement of the BCL6 protein target.

**Fig. 18:**
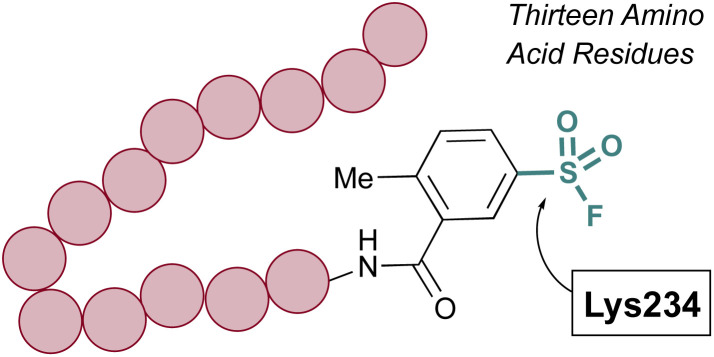
Sulfonyl fluoride-containing peptide targets Lys234 of Mcl-1.

The Pellecchia group has exploited SuFEx-based inhibition of PPIs across a variety of different systems and S(vi) fluoride electrophiles.^69,70^ A recent example includes the design of a lysine-directed sulfonyl fluoride containing BH3 peptide, as a covalent binder of the Mcl-1 protein (Fig. 18).^71^ Guided by X-ray crystal structures of a reversibly binding parent peptide interacting with Mcl-1, the group targeted a surface lysine. Here, the sulfonyl fluoride headgroup covalently anchored the PPI inhibitory peptide to the target protein, and the peptides developed were the shortest, nanomolar-potency peptide inhibitors observed to date. Additionally, the authors showed effective engagement of the peptides to the Mcl-1 protein, resulting in proteasomal-dependent degradation in a lung cancer cell line overexpressing Mcl-1.

**Fig. 19:**
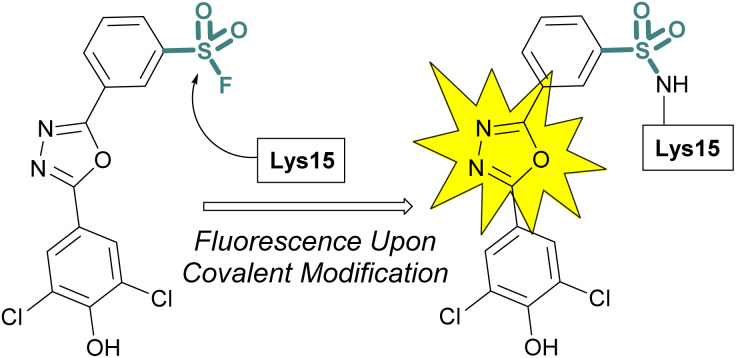
Sulfonyl fluoride covalent engagement of Lys15 leads to fluorescent turn-on of oxadiazole-based TTR inhibitors.

Sulfonyl fluoride covalent engagement can also promote the stabilization of a protein complex, as demonstrated by Kelly and coworkers, in the development of a covalent kinetic stabilizer of transthyretin (TTR, Fig. 19).^72^ Utilizing SBDD, the sulfonyl fluoride headgroup was positioned to selectively target a p*K*_a_-perturbed lysine residue within the TTR homotetramer, stabilizing the complex, and thereby preventing aggregation and the formation of amyloid fibrils. The authors postulate the importance of a glutamic acid residue in the formation of a hydrogen bonding interaction with the sulfonyl fluoride group, potentially further activating the S(vi) fluoride toward nucleophilic addition of the lysine residue. Moreover, after conjugation, several of the sulfonyl fluoride probes exhibited a fluorescence signal after covalent modification of the TTR protein complex, potentially allowing for various imaging applications to be exploited (Fig. 19).

**Fig. 20:**
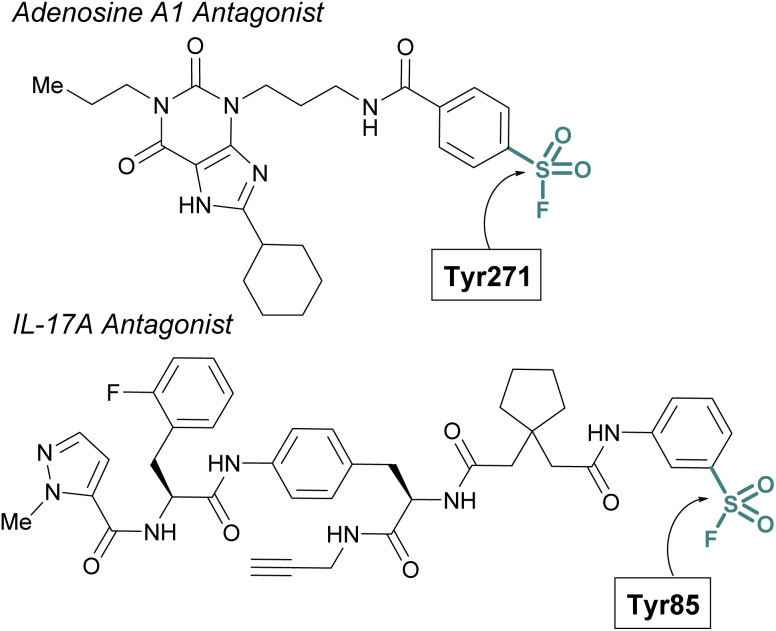
Sulfonyl fluoride probes that have enabled structural biology efforts.

Sulfur(vi) fluorides have proved enabling in structural biology efforts. Capitalizing on a known sulfonyl fluoride-containing irreversible antagonist of the A1-adenosine receptor^73^ Sexton and Christopoulos identified this probe resulted in a significant increase (∼16 °C) in the thermal stability of the protein complex.^74^ This strong stabilization was exploited to obtain a 3.2 Å crystal structure of the adenosine receptor and revealed Tyr271 was labeled by the sulfonyl fluoride. An additional example was highlighted by Liu and coworkers to aid in the confirmation of the interleukin-17A (IL-17A) antagonist X-ray structure.^75^ To solve the structure of IL-17A, the use of Fab and peptide stabilizers was required, and the team was concerned if the reversible antagonist-bound structure was an artifact. Toward this end, a sulfonyl fluoride probe was prepared that labeled the expected Tyr85, thereby confirming the validity of the reversible antagonist-bound structures and further facilitating the design of new macrocyclic analogues of IL-17A antagonists (Fig. 20).

**Fig. 21:**
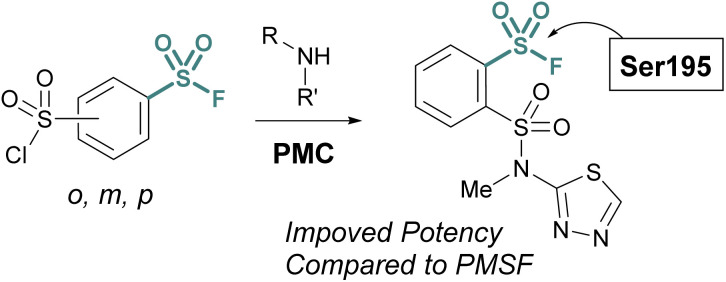
Development of PMC chemistry to access a broad array of sulfonyl fluorides leads to new inhibitors of trypsin.

Advances in synthetic and parallel medicinal chemistry (PMC) methodology to access sulfonyl fluorides, coupled with computational screening and docking studies have proved valuable in developing new sulfonyl fluoride-based probes. For example, in 2018 Grygorenko and coworkers explored the covalent docking of >3 K accessible sulfonyl fluorides against trypsin's S1 pocket, which contains an active site serine (Fig. 21).^76^ Based on their docking scores and other parameters, the top 62 compounds were then prepared and tested against trypsin in recombinant protein assays. Here, they observed three of the new inhibitors had improved IC_50_ values, as compared to PMSF, with the most potent showing a 5-fold increase in potency. Moreover, this work also showcases the stability of sulfonyl fluorides, as compared to sulfonyl chlorides in synthetic applications.

**Fig. 22:**
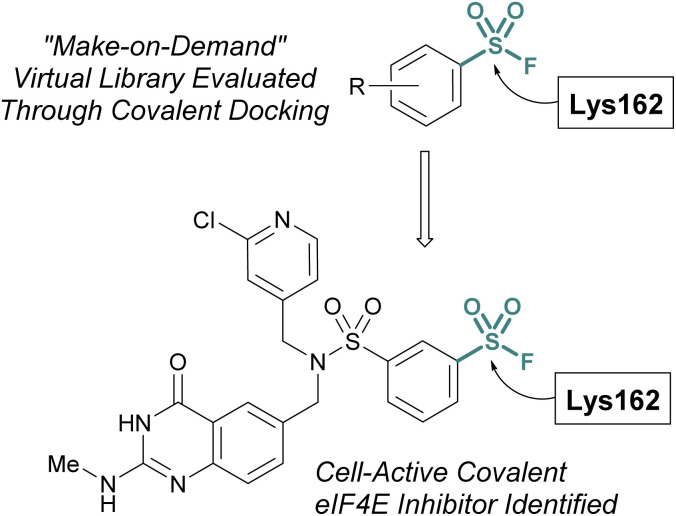
Virtual docking leads to a covalent inhibitor of elF4E targeting a lysine residue.

Additionally, the Taunton and Shoichet labs described the development of a virtual library of “make on demand” aryl sulfonyl fluorides to target the eukaryotic translation initiation factor 4E (eIF4E) protein, which has been associated with cancer cell growth and metathesis.^77^ The eIF4E protein lacks cysteine residues near the cap binding site and therefore the authors had the goal of accessing a noncatalytic lysine residue, to develop a covalent probe. Utilizing the X-ray structure of the eIF4E cap binding site, along with a covalent docking approach, ∼88 K potential sulfonyl fluoride compounds were condensed to seven that were selected for experimental evaluation of covalent binding (Fig. 22). Of these, two compounds were found to label the protein, and using this data, an additional virtual library of ∼2 K compounds were docked and evaluated. This effort identified two compounds with improved potency over the initial hit and after obtaining a co-crystal, an additional structure-based design led to the first eIF4E covalent inhibitor (*k*_inact_/*K*_i_ 0.33 μM^−1^ min^−1^) with cellular activity.

**Fig. 23:**
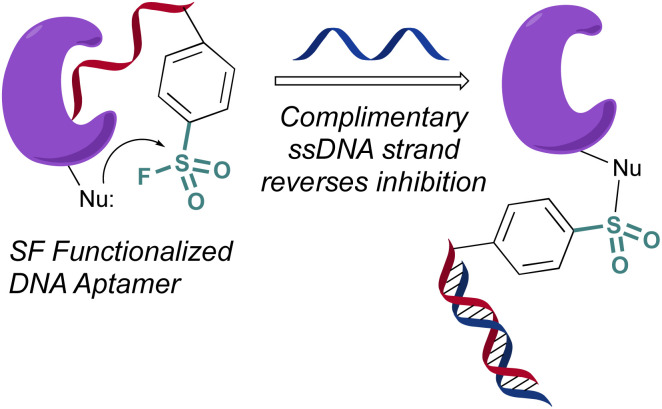
A sulfonyl fluoride appended to a DNA aptamer.

In 2020 Yang and Taki outfitted a known thrombin-binding DNA-aptamer (TBA) with a sulfonyl fluoride headgroup that covalently modified and inhibited thrombin (Fig. 23).^78^ The design positioned the sulfonyl fluoride on a linker outside the binding site of the parent TBA. This was done to not obstruct the known binding contacts but still allow for covalent modification of thrombin through a proximity-driven interaction. Toward this end, the sulfonyl fluoride-containing TBA was demonstrated to have a three-fold increase in potency over the parent TBA and covalent modification of thrombin was verified by a shift in the protein, as monitored by SDS-PAGE; however, the site of modification was challenging to access because of technical difficulties identifying the peptide-modified with the oligonucleotide. It is noteworthy that despite the irreversible interaction of the aptamer, it also possessed “on-demand reversibility” as its inhibition could be reversed by the introduction of a complementary oligonucleotide strand that sequestered the aptamer into a double strand while remaining covalently tethered to thrombin by the sulfonyl headgroup.

**Fig. 24:**
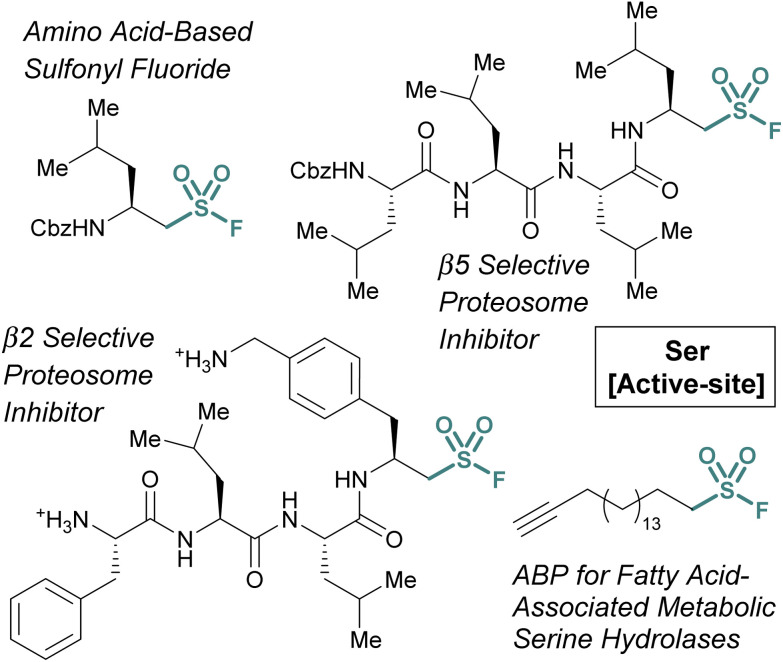
Examples of aliphatic sulfonyl fluoride for targeting active-site serine residues.

Many of the examples discussed thus far have capitalized on *aryl* sulfonyl fluorides; however, *aliphatic* sulfonyl fluorides also have demonstrated utility.^79–83^ An early example from the Liskamp group highlights the development of amino acid-derived aliphatic sulfonyl fluorides and evaluates their ability to inhibit chymotrypsin (Fig. 24).^79^ Expanding on this work, and through additional synthetic optimization, peptidic proteasome inhibitors were also developed. Utilizing the structures of known proteasomal inhibitors as their guide (*e.g.*, epoxomicin, bortezomib, and Cbz-Leu3-aldehyde), a library of elaborated sulfonyl fluoride analogues were prepared.^80^ This led to the identification of a potent compound (7 nM), that had high selectivity for the β5 subunit of the proteasome (Fig. 24). Later work showcased potent compounds with β2 selectivity over β5, in part through the incorporation of a basic amino acid residue in proximity to the sulfonyl fluoride electrophile which was accomplished through a creative synthetic strategy (Fig. 24).

**Fig. 25:**
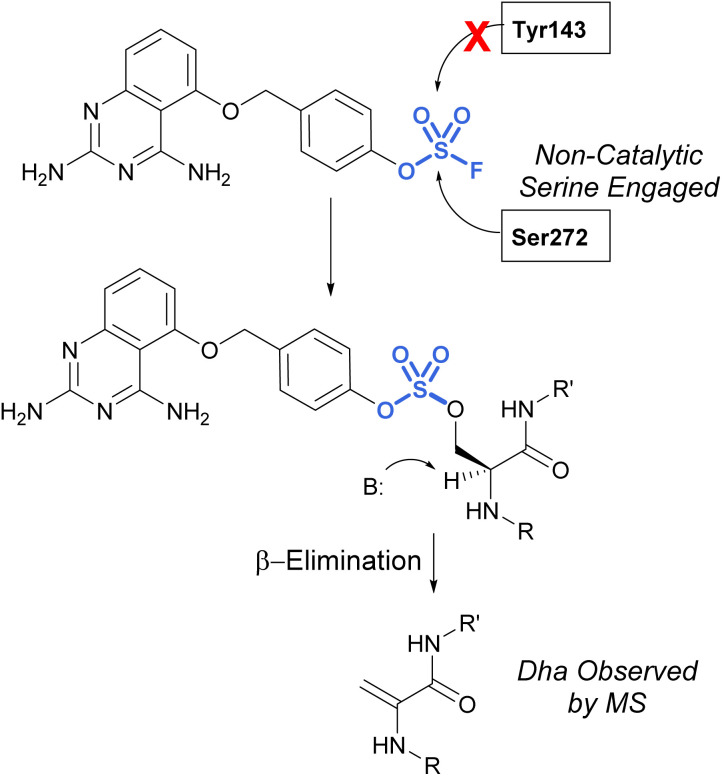
Targeting of noncatalytic serine in DcpS with a fluorosulfate moiety.

Another example of aliphatic sulfonyl fluorides was disclosed by Peng and coworkers in their report describing a fatty acid-like aliphatic sulfonyl fluoride as an ABP for a subset of fatty acid-associated metabolic serine hydrolases (Fig. 25).^84^ Here, the authors prepared clickable aliphatic sulfonyl fluorides containing either an alkyne-terminated octyl or hexadecyl (*i.e.*, OTSF and HDSF, respectively) groups and evaluated their proteomic reactivity. Treatment of HEK293 cells with the sulfonyl fluoride probes, subsequent CuAAC with rhodamine B-azide and analysis by in-gel fluorescence revealed the longer alkyl chain, HDSF probe, had more efficient labeling of proteins. Analysis of the proteins labeled by the HDSF probe was performed using stable-isotope labeling with amino acids in cell culture (SILAC)-based quantitative mass spectrometry and confirmed the covalent modification of fatty-acid-associated serine hydrolases and other fatty-acid-associated proteins at the catalytic or functionally important serine or tyrosine residues. Furthermore, the authors note that the HDSF ABP shows different proteomic reactivity from the aryl SF-containing ABP, DAS1 that was developed previously (*vide supra*).

### 3.2. Fluorosulfates

Many advancements in both medicinal chemistry and chemical biology have been made using fluorosulfates as alternative sulfur(vi) latent electrophiles. Like sulfonyl fluorides, fluorosulfates are used as electrophilic traps to covalently modify proteins, often targeting the same amino acid residues; however, fluorosulfates are chemically less reactive than sulfonyl fluorides and this has implications affecting their reactivity and selectivity observed in biological systems. This increased stability is the result of the resonance donating oxygen atom which tempers the electrophilicity of the sulfur center. Additionally, several studies compare the use of sulfonyl fluoride and fluorosulfate moieties in the context of noncovalent interactions,^85^ Tyr-targeting,^86,87^ Ser-targeting,^68,88^ Lys-targeting^89^ and His-targeting^88^ chemical probes, several of which will be discussed below.

In 2017, Fadeyi, Jones, and coworkers at Pfizer developed an aryl fluorosulfate-containing DcpS inhibitor and compared the reactivity to their previously disclosed sulfonyl fluoride DcpS probe (Fig. 25).^68,88^ First, the general reactivity of the two electrophiles was explored in an intact MS assay using recombinant human serum albumin (HSA) protein. It was observed that the sulfonyl fluoride headgroup afforded multiple protein adducts, whereas the fluorosulfate remained unreactive under the conditions explored, further showcasing the decreased reactivity of the fluorosulfate group. Furthermore, when comparing the reactivity of the probes with the DcpS protein, the fluorosulfate was observed to react with a non-catalytic serine residue in the inhibitor binding site, rather than the tyrosine residue modified by the sulfonyl fluoride-containing probe. The authors attribute the change in reactivity to the altered trajectory of the electrophilic center imparted by the additional oxygen atom in the molecule, positioning it away from the tyrosine and in proximity to the serine residue. An additional noteworthy observation was the β-elimination of the sulfate-modified serine residue to afford dehydroalanine (Dha), the major species observed in the intact MS analysis and can be used as an MS diagnostic of covalent modification. These findings emphasize not only the importance of orientation and proximity of these headgroups to nucleophilic residues in the designing of probes but also the versatility of these electrophiles in targeting nucleophiles beyond cysteine.

**Fig. 26:**
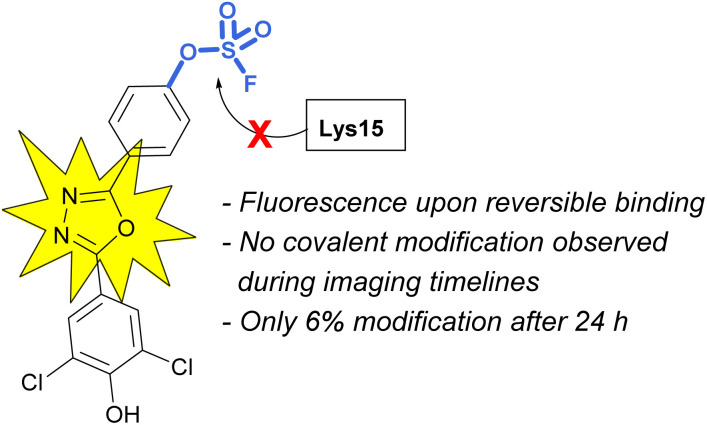
A fluorosulfate TTR inhibitor shows fluorescence turn-on with reversible protein binding.

An additional example highlighting the structural and electrophilicity differences of the sulfonyl fluoride and fluorosulfate headgroups, along with the impacts in biological applications was reported by the Kelly lab.^85^ Here, the authors compared the previously disclosed sulfonyl fluoride TTR covalent binder (Fig. 26) with similarly modified fluorosulfate-containing probes. It was found that the fluorosulfate compounds explored reacted with the target lysine only after extended incubation periods (24 h) and the modification yield was low (6%). Additionally, only the hydrolysis product (*i.e.*, the – SO_3_^−^/lysine adduct) was observed by mass spectrometry. This observed difference between the two headgroups emphasizes their differences in reactivity within the same environment. The fluorosulfate probes noncovalent binding of the TTR protein still proved valuable as there was a significant increase in fluorescence upon non-covalent probe binding and this was sufficient to allow for *in vivo* fluorescent imaging of TTR within C. *elegans*.

**Fig. 27:**
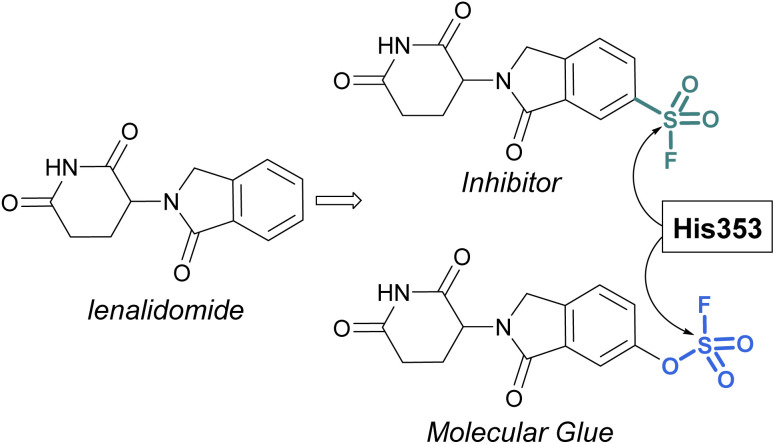
Sulfur(vi) fluorides designed to target a histidine residue on CRBN.

Sulfonyl fluorides and fluorosulfates have the capacity to covalently modify histidine residues, as recently shown by Cruite and Jones in their report describing the first rational targeting of a histidine residue by sulfur(vi) fluoride probes (Fig. 27).^90^ Guided by the X-ray crystal structure of cereblon (CRBN) bound to lenalidomide, a series of sulfur(vi) fluoride and triazole-containing lenalidomide derivatives were prepared, with the goal of engaging a specific histidine residue (*i.e.* His353). When a sulfonyl fluoride or fluorosulfate group was installed at the 6-position of lenalidomide, these compounds effectively engaged His353 (Fig. 27). Moreover, it was demonstrated that the two compounds behaved differently in live cells, with the sulfonyl fluoride probe acting as an inhibitor of the CRBN E3 ubiquitin ligase complex, thus blocking CRBN-mediated protein degradation. Alternatively, the inclusion of a single oxygen atom drastically altered the profile with the fluorosulfate probe functioning as a molecular glue and effectively degrading the protein N-terminal glutamine amidohydrolase (NTAQ1). Also of note, the authors evaluated the plasma and metabolic stability of the probes and propose that the fluorosulfate molecule has properties that may enable its use in covalent drug discovery campaigns.

**Fig. 28:**
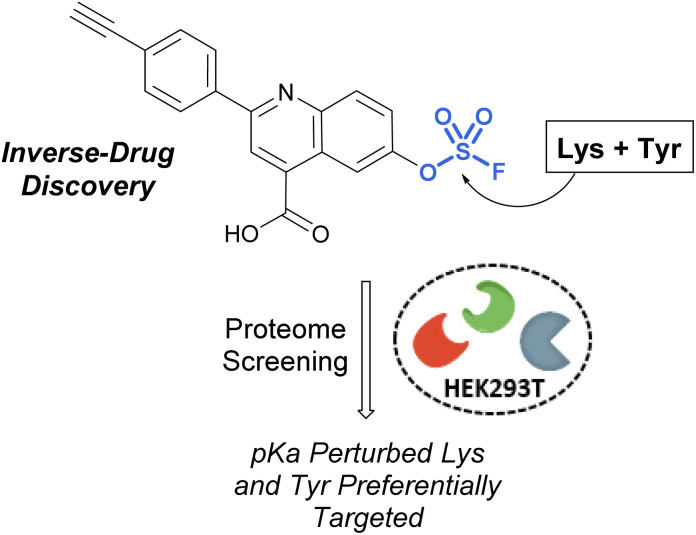
Inverse drug discovery approach utilizing fluorosulfate-containing probes.

Pairing electrophilic chemical probes with target proteins often requires extensive structural knowledge of the protein to efficiently target a specific nucleophilic amino acid residue. Conversely, Kelly *et al.* described an “Inverse Drug Discovery” strategy to identify new protein targets of fluorosulfate-containing molecules in the absence of specific protein targets (Fig. 28).^91^ This was accomplished by treating HEK293T lysates and cells with fluorosulfate-containing molecules. The inclusion of added structural complexity provided additional binding interactions and targeting of the electrophiles. An alkyne handle also enabled the pull-down and identification of covalently modified protein targets. Utilizing this approach, combined with quantitative chemoproteomics, 11 protein targets were further validated, and the reactivity of the fluorosulfate probes was demonstrated to be chemoselective toward lysine and tyrosine amino acid modifications over other amino acid residues.

**Fig. 29:**
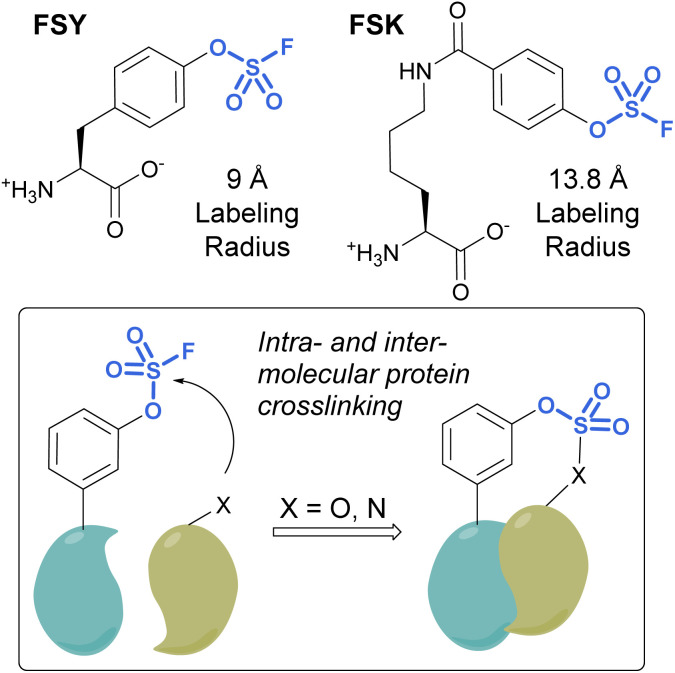
FSY and FSK are fluorosulfate-modified amino acids with different labeling radii.

Unnatural amino acids (UAAs) containing fluorosulfates have been developed and genetically incorporated into proteins of interest.^92–96^ The overall stability and context-dependent reactivity of fluorosulfates, enable the development of new electrophilic UAAs. Toward this end, Wang and coworkers synthesized a fluorosulfate-l-tyrosine (FSY) amino acid and evolved a tRNA–synthetase pair to site-selectively incorporate the UAA, both in *E. coli* and mammalian cells (Fig. 29).^92^ It was also noted that, unlike many other electrophilic UAAs, they did not observe cellular toxicity with the FSY amino acid. Moreover, the reaction of the fluorosulfate group was found to produce both intra-, as well as inter-protein crosslinking with lysine, histidine, and tyrosine residues that were proximal to FSY. A subsequent report showcased the FSY amino acid was also capable of reacting with serine and threonine residues, effectively converting these amino acids to reactive dehydroalanine and dehydrobutyrine groups on selected proteins, an approach they term Genetically Encoded Chemical Conversion (GECCO).^95^

FSY has the benefit of being similar in size to tyrosine; however, in certain instances, a larger potential labeling radius may be required for a successful reaction with amino acid residues. For this reason, Wang *et al.* also reported a new genetically encoded fluorosulfate functionalized UAA, fluorosulfonyloxybenzoyl-l-lysine (FSK), which has a longer aliphatic side chain, moving the reactive fluorosulfate further away from the protein backbone.^96^ FSY and FSK reportedly complement each other well, by making distinct covalent linkages on the same target. For example, after being genetically encoded into 7D12 nanobodies, FSY and FSK targeted different amino acid residues present on a target epidermal growth factor receptor (EGFR) (Fig. 29).

**Fig. 30:**
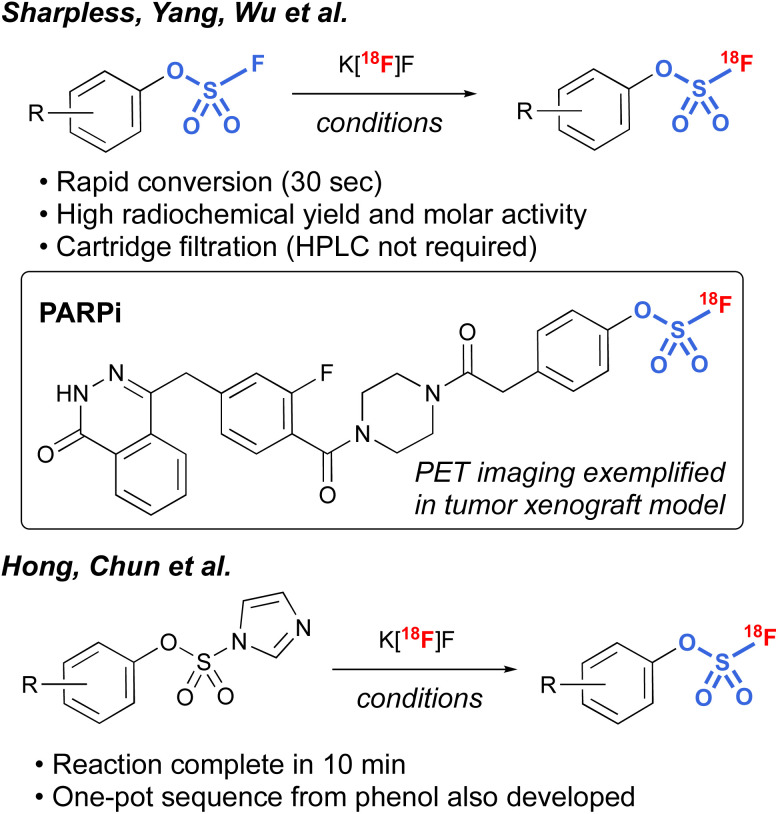
^18^[F]-labeled fluorosulfates for PET applications.

Another interesting application of fluorosulfates was demonstrated by Sharpless, Yang, and Wu *et al.*, in their development of radiolabeled fluorosulfates as [^18^F]-based positron emission tomography (PET) imaging tools.^97^ Utilizing azeotropically-dried K[^18^F]F, along with [2.2.2.] cryptand, a protocol was developed that rapidly exchanges the existing [^19^F] on the fluorosulfate moiety, resulting in conversion to the radiolabeled fluorosulfate in high radiochemical yield (RCY). It is noteworthy that the protocol was tolerant of a wide variety of different functional groups, including bioorthogonal handles (*e.g.*, *trans*-cyclooctenes and tetrazines), and only involved a cartridge filtration, thus avoiding HPLC purification that is often required in the final purification of PET imaging tools. In addition, utilizing this chemistry, a non-covalent poly(ADP-ribose) polymerase 1 (PARP1) fluorosulfate PET imaging tool compound was profiled. The synthesis of [^18^F] fluorosulfates was also demonstrated by Hong, Chun, and coworkers utilizing the radiofluorosulfurylation of aryl imidazylates, generating [^18^F^−^] labeled fluorosulfates (Fig. 30).^98^

### 3.3. Nitrogenous sulfur(vi) fluorides

**Fig. 31:**
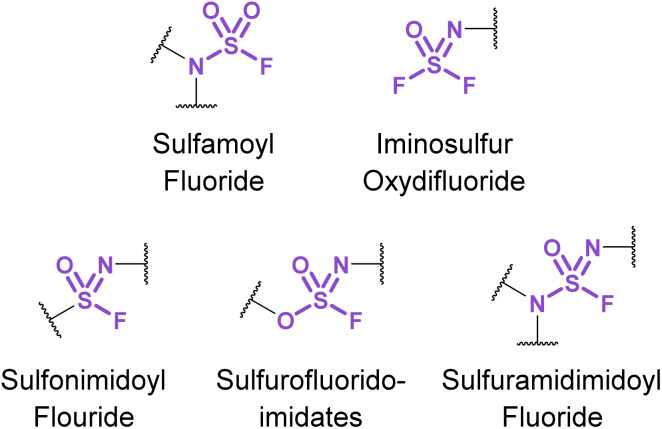
Nitrogenous S(vi) fluoride motifs.

Chemical biology and drug discovery applications of nitrogenous sulfur(vi) fluorides (Fig. 31) remain scarce, despite being structurally diverse and offering a wide range of reactivity compared to other S(vi) fluorides. The following accounts describe emerging applications of these nitrogen-based S(vi) fluorides and highlight the opportunities and challenges in developing their use.

Sulfamoyl fluorides (R_2_NSO_2_F) are nitrogenous S(vi) fluorides that have significantly different reactivities as a function of the amine group. While *N*-disubstituted sulfamoyl fluorides are relatively inert, their monosubstituted counterparts (*i.e.*, RNHSO_2_F) may readily react with nucleophiles *via* a reactive azasulfene intermediate that forms through the elimination of fluoride anion. Nevertheless, reports have demonstrated that group 2 metal compounds MgO^3^ or Ca(NTf_2_)_2_ ^99^ can be successfully employed to affect SuFEx between *N*-disubstituted sulfamoyl fluorides, amines, or alcohols. In contrast, mono-substituted sulfamoyl fluorides have been found to decompose in less than 3 minutes in a pH 7 aqueous buffer; however, in the presence of amines, can form sulfamides.^49^

**Fig. 32:**
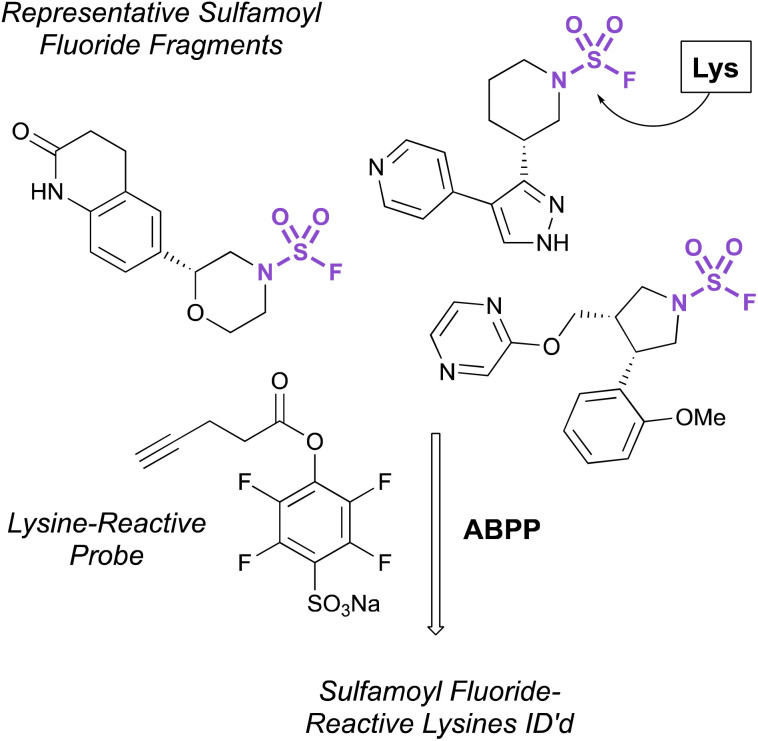
Sulfamoyl fluoride fragments for lysine modification.

Recently, the exploration of a diverse set of lysine reactive electrophiles by Abbasov, Cravatt, and coworkers shed light on the reactivity of sulfamoyl fluorides in a cellular system.^100^ In their report, 34 different chemotypes, including seven disubstituted sulfamoyl fluoride-containing fragments were evaluated for their lysine ligandability across the proteome. Utilizing a competitive ABPP workflow with a broad spectrum, lysine-reactive probe (Fig. 32), the authors were able to identify lysines that were liganded by the various aminophilic fragments. The sulfamoyl fluorides did show lysine engagement, although the overall reactivity was quite low for the fragment molecules tested compared to other sulfur-based probes (0–2 lysines were identified for each fragment among the >14 000 ligandable lysines quantified in this report).

**Fig. 33:**
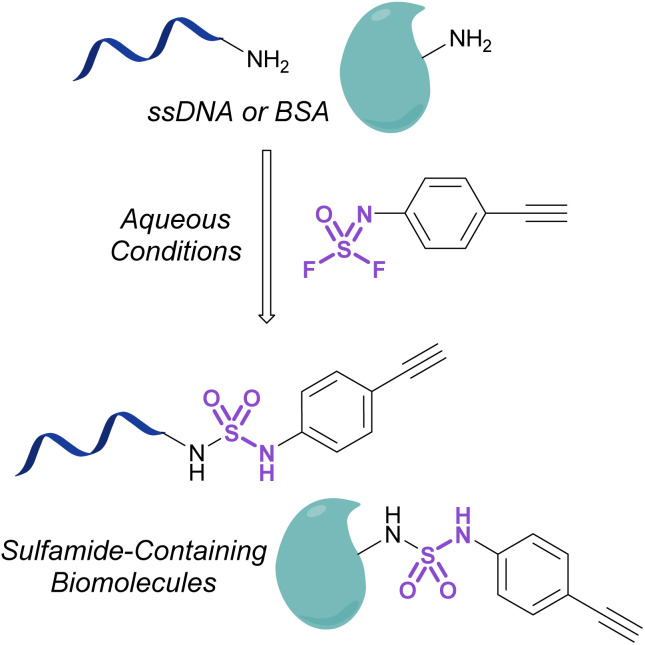
Bioconjugation of iminosulfur oxydifluoride to ssDNA and BSA.

Investigations into the utility of additional nitrogenous S(vi) fluorides in biological systems have been enabled by the development of fluorosulfurylation chemistry employing thionyl tetrafluoride (SOF_4_). Sharpless and coworkers demonstrated the value of this reactive gas in preparing the comparatively more reactive iminosulfur oxydifluorides.^101^ These nitrogenous S(vi) electrophiles were successfully demonstrated to react with both amine and alcohol nucleophiles to afford sulfuramidimidoyl fluorides and sulfurofluoridoimidates, respectively. These synthetic advances have been used to modify biologically relevant molecules such as steroids and nucleic acids with sulfonimidoyl fluorides, sulfurofluoridoimidates, and sulfuramidimidoyl fluorides, as well as in high throughput medicinal chemistry applications to rapidly generate diverse sets of molecules for screening.^52,102–105^ Moreover, Sharpless and coworkers also validated the utility of the iminosulfur oxydifluorides in bioconjugation reactions to form sulfamides on DNA and a BSA model protein under aqueous conditions (Fig. 33).

**Fig. 34:**
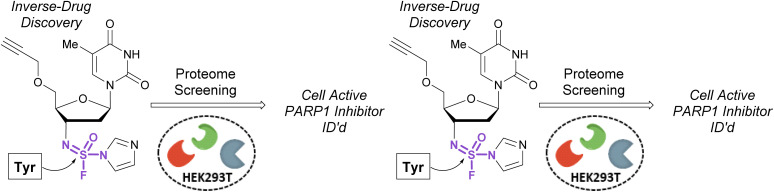
Inverse drug discovery with sulfuramidimidoyl fluorides.

In 2020, Sharpless, Kelly, and coworkers showcased the use of sulfuramidimidoyl fluorides in an ‘inverse drug discovery’ protocol, like that described in the fluorosulfate section (*vide supra*).^106^ The group treated HEK293 cell lysates with 16 structurally diverse sulfuramidimidoyl fluorides containing terminal alkyne click handles (Fig. 34). Following CuAAC with biotin azide, streptavidin enrichment, and protein digestion, the peptides were TMT labeled and analyzed by LC-MS/MS. In total, 491 distinct proteins were identified, consisting of both enzymatic and non-enzymatic proteins of which 72% were unique to the sulfuramidimidoyl fluoride class and not observed previously to react with other S(vi) fluoride electrophiles (*i.e.*, sulfonyl fluorides and fluorosulfates). Moreover, the proteins targeted varied between each sulfuramidimidoyl fluoride, depending on the structural scaffold appended to the electrophile, demonstrating the unique binding interactions necessary for protein modification. In addition, the authors found that one of their sulfuramidimidoyl electrophiles targeted PARP1, a therapeutic target for the treatment of breast and ovarian cancers. Competition, mutagenic, and LC-MS/MS analyses indicated that the electrophile covalently modifies a tyrosine 907 within the NAD^+^ binding site of PARP1. The reduced, yet tunable reactivity of S(vi)N-fluorides, along with the potential chirality associated with some analogues, makes them promising candidates for covalent inhibitors with high selectivity for the desired protein target.

## 4. Conclusion

Innovations in the synthesis and biomolecular applications of S(vi) fluorides have experienced significant growth over the past decade. Key to this work is the synergies between organic chemistry and biology – each field inspires the other. New innovations will call upon a deeper understanding of the reactivity of S(vi) fluorides both in *in vitro* and *in vivo* studies. Currently, there is no systematic understanding of how the structure and electronics of S(vi) fluorides affect the selectivity of cellular targets. These insights could provide valuable structure–activity relationships that could inform chemical biology and medicinal chemistry strategies. There is also promise in building on the existing understanding of S(vi) fluoride reactivity and metabolic stability to leverage their development in covalent drug discovery campaigns. Lastly, new strategies that enable more S(vi) fluorides to be true ‘click chemistry’ reagents would broaden the applicability of S(vi) as biomolecular tools. Unlocking this potential would be a new frontier in click and sulfur fluoride chemistry, providing exciting avenues in biomolecular chemistry.

## Conflicts of interest

J. L., T. W. B., J. D. M. and C. W. A. are employees of Pfizer.

## Supplementary Material
